# Sustainable integration of artificial intelligence and machine learning approaches within the African infectious disease vaccine research and development ecosystem

**DOI:** 10.3389/fphar.2024.1499079

**Published:** 2024-12-17

**Authors:** Jonathan Hare, Morten Nielsen, Agnes Kiragga, Daniel Ochiel

**Affiliations:** ^1^ Biolife Research Limited, Nairobi, Kenya; ^2^ Department of Health Technology, Technical University of Denmark, Lyngby, Denmark; ^3^ Data Science Program, Africa Population Health Centre, Nairobi, Kenya; ^4^ Henry Jackson Foundation Medical Research International, Nairobi, Kenya

**Keywords:** machine learning (ML), artiificial intelligence, vaccines, Africa, drug discovery

## Abstract

Artificial Intelligence and Machine Learning (AI/ML) techniques, including reverse vaccinology and predictive models, have already been applied for developing vaccine candidates for COVID-19, HIV, and Hepatitis, streamlining the vaccine development lifecycle from discovery to deployment. The application of AI and ML technologies for improving heath interventions, including drug discovery and clinical development, are expanding across Africa, particularly in South Africa, Kenya, and Nigeria. Further initiatives are required however to expand AI/ML capabilities across the continent to ensure the development of a sustainable ecosystem including enhancing the requisite knowledge base, fostering collaboration between stakeholders, ensuring robust regulatory and ethical frameworks and investment in requisite infrastructure.

## 1 Introduction

Vaccine research and development (R&D) has experienced a recent revolution following the emergence of SARS-CoV-2 as the causative agent of COVID-1), with multinational collaborative efforts to develop and deploy safe and effective vaccines globally with unprecedented speed (Hu et al., 2021). However, a second revolution in vaccine R&D is currently underway, with the incorporation of Artificial Intelligence (AI) systems and particularly the subdivision of Machine Learning (ML) algorithms positioned to provide a step change in accelerating new vaccine discoveries (Elzarrad et al., 2022; Kaushik et al., 2023) which can contribute to achieving the five key global priorities for enhancing vaccines as a tool for climate resilience as part of the recently published one health approach (Jadeja et al., 2023).

## 2 AI/ML for accelerating infectious disease vaccine discovery

The use of reverse vaccinology has revolutionized the vaccine design approach in recent times, focusing on identifying promising candidates for vaccine development through bioinformatics-based analysis of the genomics/proteomics interactions between the host and the pathogen (Patil and Shreffler, 2019). The first vaccine candidate developed using this approach was for Meningitis B and although the foundational research required took over 10 years to develop (Pizza et al., 2000; Serruto et al., 2012) it demonstrated the potential for future indications including *Streptococcus* (Prevnar 13), Herpes Zoster (Shingrix) and Malaria (Mosquirix) (Agnandji et al., 2010; Sucher et al., 2011; Lal et al., 2015). More recent studies that have incorporated reverse vaccinology and AI/ML approaches for vaccine design, include approaches for COVID-19 vaccine candidates (Bai et al., 2024; Hare et al., 2021b; Ishack and Lipner 2021; Malone et al., 2020), HIV vaccine candidates (Gaiha et al., 2019; McGowan et al., 2021), Hepatitis (Ikram et al., 2023) and *Shigella* (Aiman et al., 2023) and AMR (Khalid and Poh, 2023) amongst many others. All these vaccine candidates have been designed to include an extensive repertoire of different CD8 and CD4 T-cell epitopes to ensure that the global population is broadly covered and protected using a variety of machine-learning models (Ong et al., 2020; Reynisson et al., 2020; Hare et al., 2021a; Mazzocco et al., 2021).

Complementing cellular mediated responses are humoral responses and the quest to induce functional neutralizing antibodies through vaccination continues to present significant challenges. Examples of positive outcomes are Magar et al. that presented a machine-learning model to explore antibodies to neutralize specific SARS-CoV-2 antigens. A training dataset (VirusNet) was prepared to consist of more than nineteen hundred previously reported sequences of antigen-antibodies from several allied diseases, including HIV, influenza, SARS, and Ebola, and identified eight antibodies with the potential to neutralize COVID-19 (Magar et al., 2021). AI/ML approaches have also been utilized to redesign the variable loops of HIV-1 envelope to assist in the indication of HIV broadly neutralising antibodies (Bai et al., 2024). The applicability and functionality of many of the different AI/ML tools available for vaccine design have been summarized previously (Bravi, 2024; Olawade et al., 2024)

In addition to leveraging AI/ML to assist in the design of specific vaccines, these approaches can also be deployed to enable vaccines to be developed for variants that have yet to emerge. Thadani et al. (2023), who developed EVEscape, recently published the first iteration of this. This generalizable modular framework combines fitness predictions from a deep learning model of historical sequences with biophysical and structural information to predict pathogen variant evolution (Thadani et al., 2023). While AI/ML approaches have the power to accelerate the speed and success of vaccine discovery significantly, the power of these approaches are not just limited to the discovery space but have the potential accelerate all aspects of the development pipeline.

## 3 Utilizing AI/ML across the full vaccine development lifecycle

The vaccine development pathway has traditionally been a linear process that encompasses discovery, preclinical development through to clinical development and regulatory licensure (BMG LABTECH, 2020). Traditionally, it took 8–12 years for a product to move through this pathway. However, vaccine candidates for SARS-CoV-2 progressed through this process in 10 months (Rahman et al., 2022), although this time frame does not account for the years of technology development preceded these candidates. This expedited vaccine development pipeline can be attributed to multiple reasons, including political will, foundational research in other indications, and the transfer of failure risk from the vaccine developers to the global taxpayer (Saag, 2022; Saag, 2023).

**FIGURE 1:**
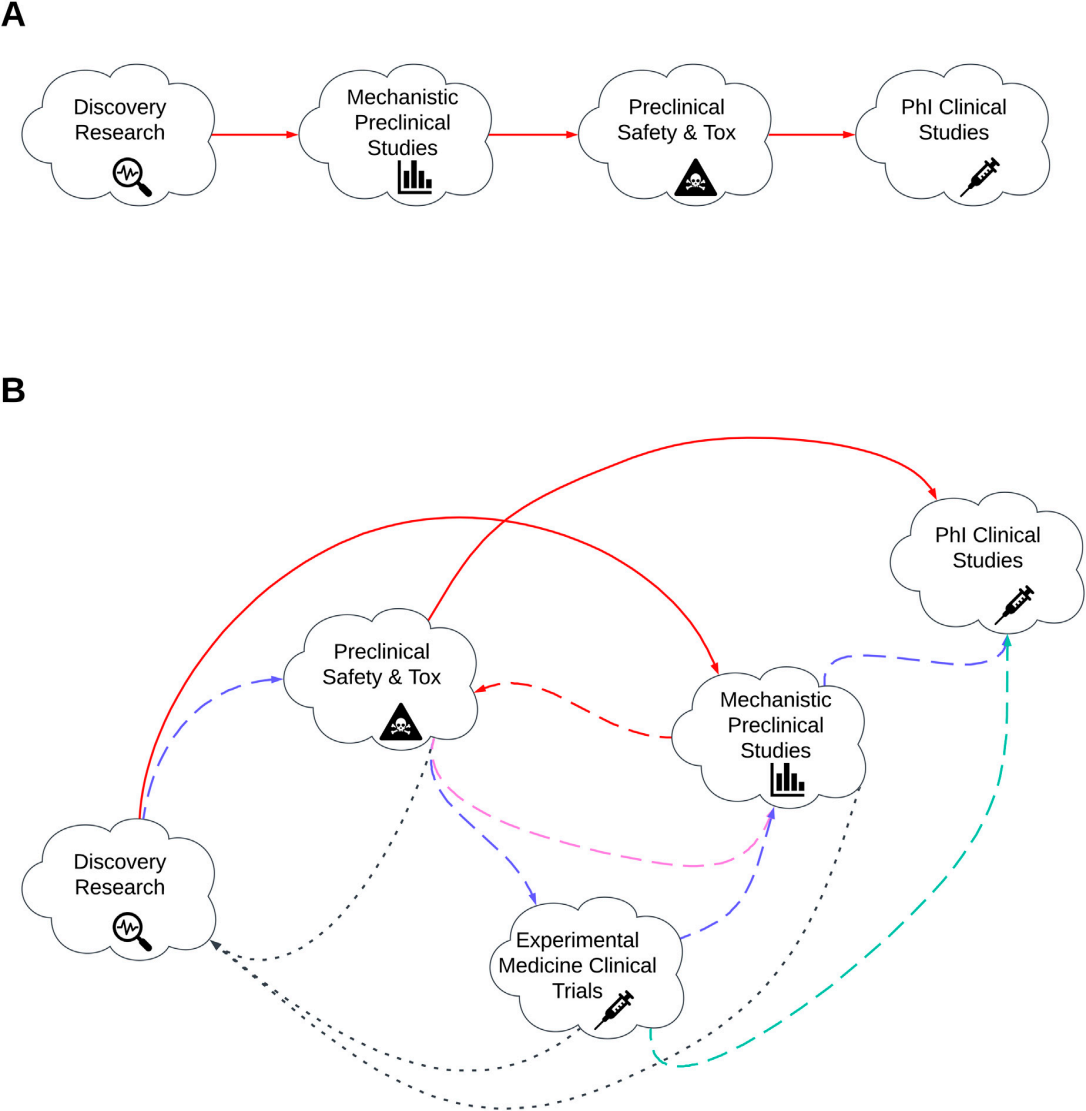
Illustration of the early phases of vaccine development. **(A)** traditional linear paradigm of vaccine development. **(B)** Reimagined vaccine development pathway. Red lines represent existing development pathway. Alternative coloured lines represent more dynamic pathway whereby by products move between clinical and clinical research phases. Blue, purple, turquoise and black represent the alternative development routes based on previous phase outcomes. Dashed lines indicate activities that progress in alignment to traditional model. Dotted lines indicate more iterative activities.

As the threat from COVID-19 recedes, the challenge moves to how best to continue with a more accelerated timeline for new product development whilst mitigating risk of failure. Lack of clinical efficacy is cited as a primary reason for the failure of Phase II clinical studies (Plenge, 2016) and highlights the need for improving target selection and developing more accurate pre-clinical models as a prerequisite for improving the success of future candidates (Herati and Wherry, 2018; Vijayan et al., 2022). The opportunity to integrate AI/ML at all phases of the vaccine development process including target selection and preclinical assessment presents an opportunity to develop a new paradigm for evaluating future vaccine candidates with greater chance of clinical success. Figure 1 illustrates the initial phases of vaccine development and covers from discovery research, preclinical assessment and in to early clinical development. Figure 1A illustrates the traditional linear paradigm whereas Figure 1B demonstrates a more integrated paradigm whereby the outputs of discovery research and early preclinical safety could be used to support initial immunogenicity testing through experimental medicine trials in humans with the data used to inform subsequent design iterations and linkage of preclinical to early clinical data.

The use of experimental medicine clinical trials and the integration of the outputs from these studies back in to target discovery and subsequent preclinical sties has been proposed previously as a solution to improve preclinical candidate selection and enable safer, cheaper, quicker and more informed developmental decision (Burt et al., 2020). Integrating AI/ML models within this paradigm should enable even greater stringency in the predictive outputs, resulting in an increased probability of developing interventions that meet thresholds for efficacy and safety.

## 4 Evaluating existing AI/ML capabilities within Africa R&D settings

AI and ML solutions have been deployed in relatively few African countries, primarily Kenya, Nigeria, Ghana, Ethiopia, and South Africa (Gadzala, 2018) focusing on the financial services, agriculture, and healthcare sectors (Ade-Ibijola and Okonkwo, 2021), though the potential benefits remain enormous with some estimates putting the potential increase to the continental GDP at over $1.5 trillion by 2030 (United Nations Development Programme, 2024).

To date, South Africa has been the leading innovator for AI adoption with a robust ecosystem offering a blueprint for other countries comprising technology hubs, academic research groups and collaborations across all sectors (Silcox et al., 2024). Recent developments in the use of AI/ML approaches to designing clinical public health interventions have led to the establishment of the Africa-Canada Artificial Intelligence and Data Innovation Consortium (ACADIC) to enable the accurate, timely, locally nuanced analysis of multiple sources of changing data to inform future clinical public health decision making (Mellado et al., 2021). There are also significant programs focusing in developing genomics research programs to expand African research capacity (de Oliveira and Baxter, 2024).

Expanding AI/ML capabilities across Africa is now underway, supported by both the private sector, government, and non-governmental organizations. Examples include a BMGF grand challenge call to develop global health and development solutions in s using AI-enabled large language models (LLMs) to expedite decision-making, policy pathways, and implementation by frontline health workers and policymakers (Announcing the 2023 Grand Challenges AI RFP Recipients Bill & Melinda Gates Foundation). A second example is an agreement between Sothema, a Moroccan pharmaceutical company and Long Island University and Baylor University in the US to expanding AI capabilities for drug discovery with expectations that the timelines for new drug development may be condensed by 60%–70% from traditional timeframes (https://www.fdiintelligence.com/content/interview/sothema-brings-ai-to-african-pharma-83386).

## 5 Strategies for sustainable deployment of AI/ML activities within the African infectious disease vaccine research and development ecosystem

The implementation of AI/ML-assisted technologies offer promising solutions to the challenges faced across Africa in vaccine R&D. These technologies have the potential to enhance efficiency, accuracy, and speed in all stages of the process spanning basic R&D in to preclinical and clinical development and beyond into manufacturing, supply chain management and post deployment surveillance. In this section we will assess how AI/ML can be deployed within these sectors offer recommendations for developing a thriving ecosystem that will foster innovation across all sectors and contribute to further elevating Africa’s vaccine R&D community.

Within vaccine discovery and preclinical testing AI/ML will accelerate the process of identifying pathogen antigen targets and their subsequent molecular and conformational structures and interactions within the selected delivery modality. By increasing the confidence that selected targets will be presented in optimal confirmation for inducing required immune responses and reducing off-target effects, AI/ML will contribute to reducing the earl phase testing. Ultimately, increasing the precision in antigen selection enhances vaccine efficacy and reduces costs by limiting failure rates.

AI/ML approaches can use used to enable optimized clinical trials designs by identifying suitable patient cohorts for particular indications that may enable more streamlined product evaluations including suitable participant identification (Hassanzadeh et al., 2020; Askin et al., 2023) and selection against particular patient characteristics, e.g., specific HLA haplotypes (Hare et al., 2021a). Coupled with assisting in trial designs, AI/ML algorithms will be able to elevate real-time data analysis during trials, facilitating more accurate decision-making and potentially reducing trial durations and improving safety profiles (Zhang et al., 2023).

The ability of AI/ML algorithms to analyse complex datasets and identify potential trends and signals within both preclinical and clinical datasets offer an opportunity to refine the process by which vaccines are tested and developing candidates in a cycle whereby clinical and preclinical testing is more integrated may ultimately results in a development timeline that is more aligned to the speed observed during the COVID-19 pandemic.

AI/ML solutions will also be crucial in supporting manufacturing, post-marketing surveillance and supply chain optimization. AI algorithms will enable the development of adaptive manufacturing processes, capable of in-life adaptations to demand variability respond to variations in population behaviours (Kumar et al., 2022). AI will also facilitate substantial improvements in real-time monitoring of vaccine safety and efficacy, enabling developers and authorities to respond rapidly to any adverse events or changing epidemiological dynamics (Ghosh et al., 2023). AI will also contribute to the optimization of supply chain logistics to ensure efficient distribution of vaccines through implementing new technological solutions (Hare et al., 2024).

The final section where AI/ML is poised to revolutionize healthcare is through personalized medicine. It is anticipated that by 2040 cancer deaths will be four times higher than current levels (Larkin, 2022) and developing expertise in personalized medicines will be essential as their use becomes more widespread as the primary oncology immunotherapeutic intervention.

## 6 Challenges and limitations

Whilst AI/ML approaches have enormous potential to evaluate the African R&D ecosystems there are certain challenges and limitations that must be addressed to ensure continued success.

The primary challenge is to develop a robust regulatory environment and associated compliance systems. Regulation of AI/ML is a hot topic, with multiple efforts ongoing to develop an enforceable regulatory framework. Within the confines of national and international agreements, it will be necessary to develop adaptive frameworks capable of responding to the rapid evolution of AI technologies. Regulatory bodies should collaborate to create a harmonized environment for innovation without compromising safety and ethical standards. Working with national and international regulators, efforts should be directed towards developing unified regulatory frameworks that facilitate the smooth integration of AI technologies into vaccine development processes and adhere to globally recognized standards.

In addition to the regulatory component there are also ethical considerations that arise when implementing AI-assisted technologies. Establishing joint ethics committees that include representatives from both AI and healthcare sectors is a critical milestone for ensuring that collaborative efforts align with the highest ethical standards. Developing robust protocols for data sharing that prioritize privacy and confidentiality is essential to mitigate ethical concerns related to patient data alongside clear ethical guidance covering additional data privacy, informed consent, and data sharing.

An additional challenge to deploying AI/ML tools for vaccine product development in Africa is the significant population diversity that exists both within countries and across the continent. Developing representative training datasets to ensure appropriate coverage will be a significant challenge and will require extensive collaboration between research groups and intergovernmental cooperation.

The final aspect to consider is the need to conduct ongoing diligence surveys of the AI landscape to ensure there is a culture of adaptability. Appropriate strategies for deploying AI to address the needs within vaccine R&D will require shifting and adapting in response to emerging challenges, and there will be a need for flexibility and a willingness to iterate on approaches to navigate this ever-evolving landscape.

## 7 Key recommendations

### 7.1 Leverage existing pan-African and global research organizations to develop strategic road map

Collaboration and synergies will be crucial in ensuring AI/ML realizes its full potential for advancing African vaccine R&D by fostering innovation, sharing expertise, and pooling resources. As a first step, it is necessary to establish a shared understanding of the challenges and gaps within the African vaccine R&D landscape and the potential of AI/ML to address core components within this ecosystem is foundational to developing a strategic plan for future success.

Key first steps will be to identify relevant stakeholders that can be drawn together to begin this process. These must include participating governments, pan-African vaccine R&D stakeholders (NGOs, R&D consortiums, African CDC) and international partners including global health organizations, funding partners and private sector partners (biotech/technology companies). The implementation of public-private partnerships can significantly contribute to resource mobilization and can foster a collective commitment to integrating AI/ML innovations within vaccine R&D.

### 7.2 Concentration of expertise

Development and retention of expertise will be key to ensuring the long-term success of any strategic plan to realize AI/ML’s potential to revolutionize the vaccine R&D landscape in Africa. There have been several consortiums initiated to advance vaccine R&D within Africa previously (Crick Africa Network, 2024; SANTHE, 2024; Kamali et al., 2015) but these have all been pan-continental consortium and have not utilized a consolidated model of centres of excellence.

To facilitate the retention of expertise and create a sustainable ecosystem that brings necessary expertise together, we propose developing regional centers of excellence focusing on applying AI/ML within the different stages of the vaccine R&D lifecycle. These centers would ideally be situated at different global transport interchanges across the continent selected for proximity to key strategic partners for supporting the selected activities. They would comprise interdisciplinary teams appropriate for the particular phase of vaccine development within the purpose-built facility.

The investment in robust infrastructure models will have additional benefits in improving connectivity and data storage to facilitate the seamless integration of AI technologies into healthcare systems. Finally, this model will eventually be regenerative, delivering programs to train and educate future scientists, researchers, and healthcare professionals in AI applications.

### 7.3 Continuous knowledge exchange

The sharing of expertise, knowledge and experience will be critical for success. Whilst the establishment of key centres of excellence should result in a vibrant ecosystem locally, mechanisms and opportunities to interact with the other centres and partner organizations will be essential; a culture of sharing is paramount.

Virtual conferences and workshops offer a platform for regular and ongoing knowledge exchange and function as a forum to delve into the latest advancements in AI, vaccine technology, and regulatory frameworks. A schedule of regular in-person meetings should also be encouraged to build relationships between colleagues.

Real-time data sharing is a crucial tool for the facilitation of novel research and the continuous enhancement of the AI/ML models. A robust data repository or secure data environment will be required for storing and sharing research findings, datasets, and algorithms. An open-source mentality should be encouraged to facilitate network transparency and sharing with the broader scientific community. However, this will require careful regulatory and ethical oversight at national and continental levels.

## 8 Conclusion and future outlook

The integration of AI/ML into vaccine development holds immense potential for transforming Africa’s vaccine R&D landscape. By strategically addressing the current challenges, fostering a culture of international collaboration, and ensuring ethical and regulatory considerations are paramount, there is the foundation for a resilient and responsive ecosystem that will propel African-led R&D to the forefront of global vaccine development.

The recommendations outlined above aim to guide policymakers, researchers, and stakeholders toward a future where AI becomes an indispensable tool in achieving these objectives. To further advance these ambitions will require urgent engagement with both global and regional stakeholders to develop the frameworks necessary to propel African-led R&D to the forefront of global vaccine development.

## Data Availability

The original contributions presented in the study are included in the article/supplementary material, further inquiries can be directed to the corresponding author.
